# Paediatric trauma mortality at a level 1 trauma centre: a retrospective case series at Karolinska University Hospital, Stockholm

**DOI:** 10.1186/s12887-026-07618-4

**Published:** 2026-09-08

**Authors:** Ammar Yaqoob, Shahzad Akram

**Affiliations:** 1 Karolinska Institutet, Stockholm, Sweden; 2 Department of Physiology and Pharmacology, Karolinska Institutet, Stockholm, Sweden; 3 Trauma Centre Karolinska, Karolinska University Hospital, Stockholm, Sweden

**Keywords:** Paediatric trauma, Trauma mortality, Penetrating injury, Interpersonal violence, Injury severity, Case series

## Abstract

Paediatric trauma mortality is uncommon in well-resourced systems, limiting clinical exposure and necessitating system-level learning through detailed review of individual deaths. This retrospective descriptive case series characterises paediatric trauma deaths following trauma-call activation at Karolinska University Hospital (KUH), Stockholm, Sweden’s primary level 1 trauma centre, between 2016 and 2025. Eligible cases were patients aged 0–17 years who triggered trauma-team activation, were entered into the Karolinska Trauma Registry, and died within 30 days of injury.

Among 1,792 paediatric trauma-call activations, 28 patients died, corresponding to a 30-day case fatality rate of 1.6% among activated trauma patients. The cohort comprised 16 males and 12 females (median age 15), and critical injury severity (New Injury Severity Score [NISS] ≥ 25) was observed in 25 of 26 cases.

Penetrating trauma accounted for most deaths (15/28), mainly stabbings and gunshot wounds; assault was recorded in half of cases, and prehospital cardiac arrest occurred in 17 patients. Isolated head injury was the most common injury pattern (12/28, 42.9%), followed by thoracoabdominal injury (4/28, 14.3%). The leading recorded causes of death were haemorrhagic shock (14/28, 50.0%) and traumatic brain injury (12/28, 42.9%); cause of death was unavailable in 2 cases (7.1%).

Fatal paediatric trauma following trauma-call activation was rare but characterised by severe injury and a substantial burden of violence-related penetrating trauma. The reported case fatality rate applies only to trauma-call activations captured in the registry and should not be interpreted as mortality among all injured children in the region. Detailed case-level review may support clinical audit, service evaluation, and prevention strategies targeting interpersonal violence among children and adolescents.

## Introduction

Injury is a leading cause of death among children and adolescents in high-income countries, although fatal paediatric trauma remains uncommon within the overall trauma population [1]. In Sweden, trauma accounts for approximately 40% of registered deaths among children aged 1–18 years, with traffic incidents, falls, and interpersonal violence as important mechanisms [2, 3].

Previous studies from major trauma centres and national registries have reported low paediatric trauma mortality in high-income settings, with blunt trauma, particularly road-traffic injury, accounting for most fatalities. Mortality is strongly associated with injury severity, traumatic brain injury, and physiological compromise on presentation. Trauma-system organisation may also influence outcomes, with evidence suggesting improved survival among injured children treated in specialised paediatric trauma centres compared with non-specialised or mixed settings [4, 5].

Despite these observations, detailed contemporary descriptions of fatal paediatric trauma remain limited, particularly within Scandinavia. Furthermore, increasing rates of interpersonal violence and penetrating trauma in Sweden may be changing the epidemiology of severe injury among children and adolescents. Detailed examination of fatal cases may therefore improve understanding of current injury patterns and inform clinical audit and public-health prevention efforts.

Children differ from adults anatomically and physiologically, and they have distinct injury patterns and clinical reserves, creating specific requirements for assessment, resuscitation, and trauma-system organisation [6]. Because fatal paediatric trauma is rare, clinical experience is limited and learning depends on detailed review of individual cases. This case series aimed to characterise all paediatric trauma deaths following trauma-call activation at Karolinska University Hospital during 2016–2025, including injury mechanism, injury intention, injury severity, prehospital characteristics, early interventions, and process times.

### Case series

This retrospective descriptive case series provides detailed case-level characterisation of paediatric trauma deaths at KUH, Stockholm, during 2016–2025, covering demographics, injury mechanism, intention, severity, prehospital variables, in-hospital interventions, and early trauma-system process times. The study was restricted to patients managed through trauma-team activation because the Karolinska Trauma Registry captures trauma-call activations rather than all injured children presenting to hospital. This registry-based cohort definition was chosen because moderately and severely injured children at KUH are routinely managed through trauma-call activation and are therefore captured in the trauma registry, whereas children with mild injuries, such as isolated simple fractures, are not included and could not be followed within the present study. Consequently, the denominator reflects patients considered to have injuries severe enough to warrant trauma-team response and does not represent all paediatric trauma presentations in the region. Trauma-call activation was based on national guidelines for paediatric trauma [7, 8]. Reporting followed principles for transparent case-series reporting, with explicit eligibility criteria, variable definitions, and complete presentation of all included cases.

### Study setting and case identification

KUH is Sweden’s primary level 1 trauma centre, receiving paediatric and adult major trauma from Stockholm and surrounding regions. Cases were identified from the Karolinska Trauma Registry, which captures all trauma-call activations using the Revised Utstein Trauma Template and is incorporated within the national SweTrau registry [9, 10]; an independent validation reported overall SweTrau data quality of 85.8% [11].

### Eligibility criteria

Cases were eligible if the patient was aged 0–17 years at the time of injury, had a trauma-call activation at KUH, and died within 30 days of injury. All eligible cases during 2016–2025 were included; patients who survived beyond 30 days were excluded. Findings should therefore be interpreted as describing mortality among paediatric trauma-call activations rather than overall paediatric trauma mortality.

### Case-series variables and definitions

Extracted variables included age, sex, dominant injury type, injury mechanism, injury intention, New Injury Severity Score (NISS), prehospital cardiac arrest (PHCA), transport modality, prehospital intubation, first emergency intervention, ventilator days, and process times from injury to hospital arrival and from emergency department arrival to first computed tomography (CT) scan or emergency intervention. Definitions followed the Revised Utstein Trauma Template where applicable. Injury intention was derived from registry coding and classified as assault, self-inflicted injury, or unintentional injury. Complete per-case data are presented in Tables 1 and 2.

**Table 1:** Baseline characteristics and injury profile of paediatric trauma deaths at Karolinska University Hospital, Stockholm, 2016–2025 (n=28)

**Case**	**Age (years)**	**Sex**	**Dominant mechanism**	**Injury mechanism**	**Intention**	**New Injury Severity Score (NISS)**	**Injury pattern**	**Cause of Death**
1	3	Female	Blunt	Traffic (pedestrian)	Unintentional	75	Crush injury thorax and abdomen	Haemorrhagic shock
2	12	Female	Penetrating	Gunshot	Assault	—	Thorax and abdomen	Haemorrhagic shock
3	2	Female	Blunt	Traffic (occupant)	Unintentional	27	Head injury	Traumatic brain injury
4	14	Female	Penetrating	Stabbing	Self-inflicted	75	Thorax	Haemorrhagic shock
5	13	Female	Blunt	Fall (high energy)	Unintentional	50	Head injury	Traumatic brain injury
6	3	Male	Penetrating	Stabbing	Assault	57	Abdomen and Head injury	Haemorrhagic shock
7	1	Female	Blunt	Fall (high energy)	Unintentional	57	Head injury	Traumatic brain injury
8	6	Male	Penetrating	Stabbing	Assault	66	Thorax and neck	Haemorrhagic shock
9	0	Female	Blunt	Unknown	Assault	24	Asphyxiation, unknown cause	-
10	11	Male	Blunt	Traffic (bicycle)	Unintentional	66	Head injury	Traumatic brain injury
11	13	Female	Blunt	Other (Hanging)	Self-inflicted	27	Asphyxiation	-
12	17	Male	Penetrating	Stabbing	Assault	41	Posterior thorax	Haemorrhagic shock
13	17	Female	Blunt	Fall (high energy)	Self-inflicted	59	Head injury	Traumatic brain injury
14	17	Male	Penetrating	Gunshot	Assault	34	Thorax and head	Traumatic brain injury
15	17	Male	Penetrating	Gunshot	Assault	75	Head and neck	Haemorrhagic shock
16	16	Male	Penetrating	Stabbing	Assault	75	Head and thorax	Traumatic brain injury
17	17	Male	Penetrating	Stabbing	Assault	51	Thorax and abdomen	Haemorrhagic shock
18	15	Female	Blunt	Traffic (occupant)	Unintentional	43	Head injury	Traumatic brain injury
19	17	Female	Blunt	Fall (high energy) + Burns	Self-inflicted	48	Head injury	Traumatic brain injury
20	15	Male	Penetrating	Stabbing	Assault	75	Thorax and abdomen	Haemorrhagic shock
21	15	Male	Penetrating	Stabbing	Assault	75	Thorax	Haemorrhagic shock
22	15	Male	Blunt	Traffic (occupant)	Self-inflicted	59	Head injury	Traumatic brain injury
23	17	Male	Penetrating	Gunshot	Assault	—	Thorax and abdomen	Haemorrhagic shock
24	16	Male	Blunt	Fall (high energy)	Self-inflicted	50	Head injury	Traumatic brain injury
25	16	Male	Penetrating	Gunshot	Assault	75	Posterior thorax	Haemorrhagic shock
26	17	Female	Blunt	Traffic (pedestrian)	Unintentional	66	Head injury	Traumatic brain injury
27	15	Male	Penetrating	Blast (Firework)	Unintentional	75	Head injury	Traumatic brain injury
28	16	Male	Penetrating	Gunshot	Assault	54	Head injury	Traumatic brain injury

Cases are presented in randomised order. Variable definitions follow the Revised Utstein Trauma Template v1.1.1. Dominant mechanism indicates the injury type with the highest Abbreviated Injury Scale score: blunt injury refers to diffuse force, and penetrating injury refers to tissue penetration by a sharp or projectile object. Injury mechanism denotes the external cause of injury. Injury intention denotes suspected intent: unintentional, self-inflicted, or assault. NISS = New Injury Severity Score, range 0–75; NISS ≥25 indicates critical injury severity. Injury pattern denotes the predominant anatomical region or regions injured. Cause of death describes the predominant cause leading to death. — indicates missing or unavailable registry data

**Table 2:** Prehospital and early in-hospital process variables among paediatric trauma deaths at Karolinska University Hospital, Stockholm, 2016–2025 (n=28)

**Case**	**Injury mechanism**	**Pre-hospital time (min)**	**Pre-hospital cardiac arrest**	**Transport**	**Pre-hospital intubation**	**Time to intervention (min)**	**Computed tomography / intervention **	**Intervention type**
1	Traffic (pedestrian)	68	Yes	Helicopter emergency medical services (HEMS)	Yes	0	Intervention	Damage Control laparotomy
2	Gunshot	31	No	Ground ambulance	Yes	7	Intervention	Damage Control thoracotomy
3	Traffic (occupant)	42	No	HEMS	Yes	36	CT	Intra-cranial pressure device
4	Stabbing	—	Yes	HEMS	Yes	—	—	—
5	Fall (high energy)	61	No	HEMS	Yes	21	CT	—
6	Stabbing	55	Yes	HEMS	No	17	Intervention	Damage Control thoracotomy
7	Fall (high energy)	52	—	—	—	38	CT	Intra-cranial pressure device
8	Stabbing	25	Yes	Ground ambulance	Yes	0	Intervention	Damage Control thoracotomy
9	Unknown	101	Yes	HEMS	Yes	—	—	—
10	Traffic (bicycle)	33	Yes	HEMS	Yes	8	Intervention	Damage Control thoracotomy
11	Other	48	Yes	Ground ambulance	Yes	—	—	—
12	Stabbing	32	Yes	Ground ambulance	Yes	2	Intervention	Damage Control thoracotomy
13	Fall (high energy)	29	No	HEMS	Yes	19	CT	Craniotomy
14	Gunshot	38	Yes	Ground ambulance	Yes	1	Intervention	Damage Control thoracotomy
15	Gunshot	42	Yes	Ground ambulance	Yes	30	CT	—
16	Stabbing	46	Yes	HEMS	Yes	—	—	—
17	Stabbing	33	Yes	Ground ambulance	Yes	0	Intervention	Damage Control thoracotomy
18	Traffic (occupant)	21	No	Ground ambulance	No	—	—	—
19	Fall (high energy)	58	Yes	Ground ambulance	Yes	—	—	—
20	Stabbing	30	Yes	Ground ambulance	Yes	3	Intervention	Damage Control thoracotomy
21	Stabbing	33	Yes	Ground ambulance	Yes	—	—	—
22	Traffic (occupant)	44	No	HEMS	No	22	CT	Craniotomy
23	Gunshot	23	No	Ground ambulance	No	2	Intervention	Damage Control thoracotomy
24	Fall (high energy)	26	Yes	HEMS	Yes	10	Intervention	Other
25	Gunshot	22	Yes	Ground ambulance	Yes	5	Intervention	Damage Control thoracotomy
26	Traffic (pedestrian)	25	No	HEMS	Yes	22	CT	Craniotomy
27	Blast	57	No	Ground ambulance	Yes	32	CT	Other
28	Gunshot	27	No	HEMS	Yes	13	CT	Craniotomy

Cases are shown in randomised order and correspond to the same case numbers as in Table 1. Variable definitions follow the Revised Utstein Trauma Template v1.1.1. Prehospital time is the interval from injury to hospital arrival, reported in minutes. Prehospital cardiac arrest refers to injury-related cessation of cardiac activity with absent pulse, unresponsiveness, and apnoea. Transport by helicopter emergency medical services denotes helicopter-based emergency medical transport. Prehospital intubation refers to airway management with an adjunct device before hospital arrival. Time to intervention is the interval from emergency department arrival to either the first computed tomography scan or the first key emergency intervention, whichever occurred first. Computed tomography indicates that imaging was performed first, whereas intervention indicates that an emergency procedure was performed first. Intervention type refers to the first key emergency intervention recorded for treatment or stabilisation. — indicates missing, unavailable, or not applicable data

### Mortality review

All fatal cases were reviewed during routine multidisciplinary mortality meetings conducted as part of local and national trauma quality assurance and held monthly. The review group included trauma surgeons, emergency physicians, anaesthesiologists, intensivists, radiologists, paediatric surgeons, prehospital clinicians when relevant, trauma coordinators, and registry staff. Reviews were based on available prehospital records, emergency department documentation, imaging findings, operative reports, intensive-care documentation, and registry data. The purpose was to identify potential areas of improvement in trauma management. No deaths were considered potentially preventable during local review.

### Ethical considerations

All data were anonymised before analysis, and no personal identifiers are included in this manuscript. Ethical approval was granted by the Swedish Ethical Review Authority, diary number 2024-03805-01, with addendum 2025-08751-02.

## Results

### Cohort characteristics

Of 1,792 paediatric trauma-call activations at KUH during the study period, 28 met the inclusion criteria (30-day case fatality rate 1.6%). The cohort comprised males (*n* = 16, 57.1%) and females (*n* = 12, 42.9%); age range was 0–17 years (median 15, mean 12.6, SD 5.7).

### Injury mechanism, intention, and severity

Penetrating trauma (*n* = 15, 53.6%) comprised stabbings (*n* = 8), gunshot wounds (*n* = 6), and blast injury (*n* = 1). Blunt trauma (*n* = 13, 46.4%) comprised high-energy falls (*n* = 5), traffic incidents (*n* = 6; vehicle occupants *n* = 3, pedestrians *n* = 2, cyclist *n* = 1), unspecified blunt trauma (*n* = 1), and other mechanisms (*n* = 1). Injury intention was recorded as assault (*n* = 14, 50.0%), unintentional injury (*n* = 8, 28.6%), or self-inflicted injury (*n* = 6, 21.4%). NISS was available in 26 of 28 cases; 25 of these had critical injury severity (NISS ≥ 25) and 19 had NISS ≥ 50. The single case with NISS 24 involved blunt trauma of unknown mechanism, with assault as the recorded intention. All cases were reviewed during routine multidisciplinary peer-review mortality meetings, as mentioned above. No cases of potentially preventable death were identified.

### Prehospital characteristics and emergency interventions

Prehospital cardiac arrest (*n* = 17, 60.7%) and prehospital intubation (*n* = 23, 82.1%) were common. Transport was by ground ambulance (*n* = 14, 50.0%) or helicopter emergency medical services (*n* = 13, 46.4%), with transport modality unavailable in 1 case. Damage-control thoracotomy was the most frequent first emergency intervention (*n* = 10, 35.7%), followed by craniotomy (*n* = 4), intracranial pressure monitor insertion (*n* = 2), damage-control laparotomy (*n* = 1), and other interventions (*n* = 2). No key emergency intervention was recorded in 9 cases (32.1%), reflecting death before a procedure, initial management with computed tomography and intensive care, or incomplete documentation.

### Process times

Time from injury to hospital arrival was available in 27 of 28 cases, with a median of 33 min (range 21–101). Among cases in which CT was the first recorded in-hospital event, median time from emergency department arrival to CT was 22 min (range 13–38; *n* = 9). Among cases receiving an emergency intervention as the first recorded event, median time from emergency department arrival to first procedure was 2.5 min (range 0–17; *n* = 12).

### Injury patterns and causes of death

Among recorded injury patterns, isolated head injury was most common (*n* = 12, 42.9%), followed by thoracoabdominal injury (*n* = 4, 14.3%). Thoracic-only injury, posterior thoracic injury, combined head-and-thorax injury, and asphyxiation-related presentations each occurred in 2 cases (7.1%). Less frequent patterns included crush injury involving the thorax and abdomen, combined abdominal and head injury, thorax-and-neck injury, and head-and-neck injury, each occurring in 1 case (3.6%). The leading recorded causes of death were haemorrhagic shock (*n* = 14, 50.0%) and traumatic brain injury (*n* = 12, 42.9%).

### Missing data

NISS was unavailable in two patients (7.1%), transport modality in one patient (3.6%), and prehospital transport-time data in one patient (3.6%). Cause of death was unavailable in 2 patients (7.1%).

In-hospital timing variables and intervention details were not applicable or unavailable in several patients who died before definitive imaging or procedure, or where registry documentation was incomplete. Given the small cohort, even limited missing data may affect interpretation of case-level patterns; therefore, all analyses are presented descriptively and without inferential comparison.

## Discussion

This 10-year case series identified 28 paediatric trauma deaths at a high-volume national trauma centre, underscoring the rarity of fatal paediatric trauma in a well-resourced system and the value of case-level analysis when numbers are too small for inferential comparison. The 30-day case fatality rate of 1.6% is consistent with European data, with comparable rates of 1.4% at a major Danish trauma centre [12] and 2.8% in the Dutch national trauma registry [13]. The male predominance (57.1%) is also consistent with paediatric trauma mortality series.

The principal distinction from comparable European series was the mechanism of death. Scandinavian and Northern European studies generally identify blunt trauma, particularly road-traffic injury, as the leading contributor to paediatric trauma mortality; traffic-related trauma accounted for 46.5% of fatal cases in the Dutch series [13], and blunt mechanisms predominated across all severity strata in the Danish series [12]. By contrast, more than half of deaths at KUH involved penetrating trauma. This pattern indicates a local concentration of severe violence-related injury among adolescents, without allowing conclusions about trends, causes, or trauma management. Critical injury severity nonetheless appears consistent across settings, with more than 90% of fatal cases in both European cohorts classified as critically injured [12, 13].

The high proportion of assault-related deaths (*n* = 14, 50.0%) mirrors emerging national trends in Sweden, where severe interpersonal violence and penetrating trauma have increased, particularly among adolescents affected by firearm- and gang-related violence [14, 15]. The registry lacks socioeconomic, neighbourhood, and ethnicity data, limiting analysis of vulnerability factors; nonetheless the findings support viewing fatal paediatric trauma as a regional public health and prevention challenge, as well as a clinical one.

The anatomical injury patterns and causes of death provide further context for these findings. Isolated head injury was the most common recorded injury pattern (42.9%), and traumatic brain injury accounted for 42.9% of deaths. This is consistent with paediatric trauma literature identifying traumatic brain injury as a major driver of trauma-related mortality and disability in children [16, 17]. However, haemorrhagic shock was the leading recorded cause of death in the present series (50.0%), reflecting the high proportion of penetrating thoracic, abdominal, and combined torso injuries. This differs from many predominantly blunt paediatric trauma cohorts, where traumatic brain injury and road-traffic mechanisms often dominate, and supports the interpretation that the KUH series represents a local pattern of severe violence-related injury rather than a typical all-cause paediatric trauma mortality distribution [12–14].

The present case series was intentionally limited to fatal cases. A direct comparison with the broader paediatric trauma population treated at KUH would require inclusion of non-fatal trauma-call activations and children not captured by the trauma registry. Such an analysis is outside the scope of this manuscript but is important for future work, particularly to distinguish features specific to fatal outcomes from characteristics common among severely injured children who survive.

The observed burden of violence-related penetrating injury highlights the importance of prevention strategies beyond the healthcare setting. Potential approaches include school-based violence-prevention programmes, early intervention for at-risk youth, collaboration between healthcare services, schools, social services, and law-enforcement agencies, and enhanced regional surveillance systems capable of monitoring trends in violence-related injury. Such measures may complement trauma-system improvements by addressing upstream determinants of severe injury among children and adolescents.

Several limitations should be considered. The registry includes only children who triggered level 1 or level 2 trauma-call activation according to national guidelines; consequently, children with minor injuries who did not require trauma-team response were not captured. In addition, the small number of deaths limited subgroup analyses and precluded robust comparative assessment.

## Conclusion

In this 10-year series from Sweden’s primary level 1 trauma centre, fatal paediatric trauma following trauma-call activation was rare but marked by extreme injury severity, frequent prehospital cardiac arrest, and a predominance of violence-related penetrating injury. Isolated head injury was the most common injury pattern, yet haemorrhagic shock was the leading recorded cause of death, followed closely by traumatic brain injury. These findings suggest a fatal injury profile shaped by both severe neurotrauma and exsanguinating torso trauma. Detailed case-level review should be integrated into ongoing trauma audits, while the burden of interpersonal violence among children highlights the need for prevention extending beyond hospital-based care in the greater Stockholm Region. 

## Data Availability

Data is available through the Karolinska Trauma Registry and SweTrau.

## Abbreviations

CT: Computed Tomography
ED: Emergency Department
HEMS: Helicopter Emergency Medical Services
ICP: Intracranial Pressure
PHCA: Prehospital Cardiac Arrest
KUH: Karolinska University Hospital
NISS: New Injury Severity Score
